# Relaunching arcade games: game nostalgia and collective memory practice for China’s first-generation players

**DOI:** 10.3389/fpsyg.2026.1784510

**Published:** 2026-04-14

**Authors:** Junxin Li, Ziran Zhao, Jiajie Guo

**Affiliations:** 1 School of Journalism and Communication, Chongqing University, Chongqing, China; 2 School of Arts and Social Sciences, Monash University, Melbourne, VIC, Australia; 3 School of Literature and Journalism, Sichuan University, Chengdu, China

**Keywords:** arcade, China, collective memory, first-generation players, nostalgic consumption

## Abstract

This study examines the arcade memory of the first-generation players in China through semi-structured interviews. Using Menke’s framework of media nostalgia as an analytical tool, this study found that the narrative of arcade memory is formed through game text, material technology and spatial structure: Players established a visualized memory mode by revisiting the game world and recalling the material sensations generated through human–machine interaction; the coexistence and interactions among multiple actants shape the arcade hall as a landscape of public memory and spatial affect. The memory of arcade games emerges as a geographical and interest-based collective memory centered on weakly connected neighborhood life and generational identity. By analyzing the memory practices surrounding arcade games, this study develops a theoretical framework for understanding game nostalgia, thereby contributing to the broader fields of game studies and memory studies.

## 1 Introduction

The harsh timbre of electronic synthesized sounds mixed with the clatter of joysticks and onlookers’ cheers, as the glow of colorful pixelated graphics intersected with the dim lighting of arcade halls. The interplay of these soundscapes and lightscapes surrounding arcade machines has become a cue to nostalgia, cultivating memory practices through which players pursue gaming retro aesthetic (Collins, 2016; Niemeyer, 2014). The arcade refers to both coin-operated video game machines and the venues in which they operate, before video games entered domestic spaces via consoles or mobile devices, arcades occupied urban public spaces as entertainment infrastructure (Banfi, 2024). Embedded within localities such as migrant housing districts and amusement halls, arcades became key sites for sustaining self-identity and shaping collective belonging. They enabled individuals to negotiate the uncertainties of technological change, bridging the affective gap between the excitement of new technologies and the stability symbolized by older ones (Imai and Woite, 2024).

In China, arcade games entered the market in the late 1980s and early 1990s, coinciding with the country’s *Reform and Opening*. As an interactive entertainment medium, arcade games swiftly permeated urban public spaces (Zhang, 2013). Young people entered these game worlds by inserting coins, forming both the lifestyle and consumer culture of the early technological era among those “first generation” players. With the economic boom of the 1990s, arcades flourished in major Chinese cities. Alongside imported fighting games such as *Street Fighter* and *The King of Fighters*, domestically developed titles that incorporated Chinese classics and national elements also emerged, such as *Xi You Shi E Zhuan* (西游释厄传) and *San Guo Zhan Ji* (三国战纪). However, this golden era was accompanied by social controversies, including anxieties over cultural dependence on foreign brands and associations with gambling and violence (Wang, 2018; Zhang, 2013). State regulation consequently tightened, leading to mass crackdowns and closures of arcades, education authorities went further, even mobilizing students to sign pledges to quit gaming, framing video games as spiritual opium, thereby deepening the stigmatization of arcades (Zhou, 2024).

By the early 21st century, the rise of the internet, personal computers, and smartphones eroded the arcades’ former market dominance. Nonetheless, arcades did not disappear entirely; rather, they came to serve as symbols of a past era. They constitute sites of memory centered on technological culture and entertainment spaces, offering a unique lens through which to examine tensions between generational memory, media governance, and consumer practices (Wolf, 2003). As Sturken (2008) reminds us, memory studies cannot overlook nostalgic practices connected to everyday experiences and popular commodity culture. Nostalgia for the arcade is not only a crucial lens for understanding the development of contemporary media technologies and gaming cultures, but also a reenactment of 1990s Chinese leisure practices that shaped the emotional structures of player communities. Participation in arcade gaming was conditioned by a constellation of actors (developers, venue operators, players), institutions, and environments (Litherland, 2021). Moreover, arcades extended into new forms of social performance, establishing an ecosystem that included players, onlookers, and fans. Within this ecology, participants negotiated social and cultural conventions, constructing and performing subjectivities (Skolnik and Conway, 2019). Thus, any study of arcade memory practices must analyze discourses, forms, and practices of memory while attending to their entanglements with generational experiences and collective affect.

Existing scholarship on arcades has largely focused on game ontology and historical trajectories (Banfi, 2024; Borowy, 2013), regulatory policies (Zhou, 2024), urban landscapes (Imai and Woite, 2024; Kidokoro et al., 2022; Skolnik and Conway, 2019), player transactions/social practices (Lin and Sun, 2011; Zalot, 2023), and broader social impacts (Sawers and Demetrious, 2010). But only few studies have examined arcades in relation to nostalgic practices. Moreover, the geographical emphasis has remained on Europe and North America (Kocurek, 2015; Litherland, 2021; Meades, 2022), with limited attention to the specific trajectories of arcades in China. Building on Menke’s (2017) theoretical frameworks of media nostalgia, this study addresses the following questions: How have “first-generation” players engaged in nostalgic practices around arcades? How have such practices shaped their affective structures? How do collective memories of arcades inform contemporary gaming culture and consumption? By interpreting and reflecting on data we collected, this article delineates the material characteristics of arcade media and the processes of memory and meaning-making within the gaming practices of a generation.

## 2 Media nostalgia on games

This article adopts Menke’s (2017) concept of media nostalgia as the core framework to examine nostalgic practices in game memory. Media nostalgia refers to a longing for and retrospection on past media cultures and technologies. It is not merely an expression of individual emotion, but a collective cultural phenomenon reactivated within the representation-reproduction modes of modern media (Kappes and Menke, 2024). Unlike mediated nostalgia, in which media serve as intermediaries for remembering, media nostalgia takes the medium itself as the object of nostalgia (Lizardi, 2015; Niemeyer, 2014). It is otherwise to say, media artifacts and apparatuses become cherished objects not only because of what they contain, but also because of their own biographical value, linking material forms to both memory and present affection (Kappes and Menke, 2024).

Media nostalgia is rooted in personal emotional recollections of lost time and objects, yet it also transforms into collective cultural practice through shared media codes, signifiers, and motifs (Boym, 2001; Pickering and Keightley, 2006). At the collective level, it constructs a representation of the past that transcends generations and communities, making nostalgia an important mechanism for negotiating social memory and cultural identity (Kalinina and Menke, 2016). Nostalgia emerges from the irretrievability of the past time, the loss of childhood, and dissatisfaction with the present (Massantini, 2020). As such, media nostalgia reflects modes of perception and attitudes toward the contemporary sociocultural landscape (Radstone, 2010).

According to Menke (2017), media nostalgia encompasses three dimensions: media content (the representational or mediated content), media artifacts (the material objects or data-storage media as carriers of information), and media apparatuses (the hardware and technologies that reproduce information). In the context of game nostalgia, media content corresponds to retrogaming, in which games are remade or remastered from classic titles of the 1970s to 1990s in terms of aesthetics, technology, and marketing. Through such commodified forms of media, memory culture becomes commercialized (Whalen and Taylor, 2008). Media artifacts correspond to players’ gaming experiences as memory subjects. Through revisiting their play histories, players construct narrative forms of memory, for instance, by using “save files” as material archives of lived experience, or by engaging in restorative and reflective nostalgic practices within the game world. Media apparatuses refers to the material memories of gaming consoles, whose nostalgic value drives from the collection of historically significant technologies. As Guins (2014) argues, video games are total objects that encompass software, hardware, and specific spaces; memory of games is thus grounded in the embodied experience of operating game machines. With the continual evolution of media technologies, old consoles have gradually become collectibles that embody players’ childhood memories (Wolf, 2008).

Just as media nostalgia foregrounds human–media interactions, games not only shape players’ cultural reflections but also construct new ways of remembering the past. Current research on game memory primarily focuses on how games represent past eras, that is, the level of media content: games are regarded as emergent narrative media that interpret historical events and processes (Hess, 2007). Scholars of historical games, such as Chapman et al. (2016), argue that games can re-present the past by constructing historical locations, architectures, and characters, as well as by simulating the unfolding of events. Through such design, games become an environment for experimentation, enabling players to explore and negotiate discourses of history (Zhao et al., 2025). In this sense, games constitute participatory sites of memory, where simulation and reconstruction of history generate new modes of remembering (Kapell and Elliott, 2013).

However, research on player memory cannot be confined to the level of media content. Kappes and Menke’s (2024) practice-oriented view of media nostalgia emphasizes that nostalgic practice is realized only through the body and retrospective reflection of the user. Hence, media as objects of use are as indispensable as the bodily and mental activities that engage them (Reckwitz, 2002). This practice-theoretical orientation, which treats the medium itself–including interactive objects and their products–as an actor, shifts attention to what users do and experience in everyday contexts, emphasizing bottom-up practices. Arcades and arcade halls were vital public entertainment spaces in the past, they exemplify this shift. Investigating players’ nostalgic engagement with arcade games thus requires attention not only to the game text but also to the meanings and experiences produced through embodied interactivity, as well as to players’ inter-group interactions and lifestyles within specific social environments. This article examines the nostalgic practices and collective memories of China’s “first-generation” arcade players in the 1990s, situating the study of game memory within the Chinese sociocultural context. In doing so, it contributes new perspectives and practical agendas to the study of media and collective memory.

## 3 Method and data

This study adopts the research perspective of how people use media to recall the past, using semi-structured interviews to collect empirical materials. The interviews were mainly conducted through open recruitment on popular Chinese social media and gaming communities: The interviewees were mainly from the “Mutual Memory Group” on *Douban*, the “Arcade Game Bar” on *Baidu Tieba*, columnists of “Arcade Era” on *Zhihu*, as well as social media platforms like *Weibo* and *Rednotes*. Online communities such as *Douban* Groups, *Baidu Tieba*, and *Zhihu* are centered around interest-based aggregation. They provide authentic and tangible discursive material for studying the construction of collective memory and the expression of nostalgic sentiment, allowing for a clear capture of the formation logic, dissemination paths, and connections to the contemporary context of collective memory. For instance, the “Mutual Memory Group” on the *Douban* has over 230,000 members; the “Arcade Game Bar” on *Baidu Tieba* has 154,000 members and 96,000 posts; and the “Arcade Era” on *Zhihu* has more than 110,000 comments and 170 million views. These communities are highly interactive, with members primarily collaborating in the form of shared memory to collectively recall information. Through exchanges and discussions, they help each other reactivate and establish memory cues. Participants were recruited using a combination of convenience and snowball sampling methods. In addition, the research team conducted informal visits to retro arcades in contemporary cities to better understand the nostalgic consumption scenes described by the interviewees, thereby providing contextual background for the interpretation of the interview data.

The interviews ultimately involved 18 participants, with the interview period concentrated from September to December 2024. Each player’s interview was conducted in 2–3 sessions, and the total interview time for each individual player was approximately 60 min. The subjects of this study are arcade game players from the 1990s, and the ages of the participants in the interviews range from 34 to 48 years old, with a majority being male players (16 male and 2 female), because in the 1990s, arcades in China were primarily spaces frequented by men (Chen et al., 2023). The study also included 2 offline interview groups, each consisting of 2–3 players, to create an environment for sharing memories. The interview numbers were mainly arranged in alphabetical order of the first letter of the participants’ names, and for those with the same first letter, they were listed in chronological order of the interview time as 1, 2, 3, etc. The theme of the interviews was “Memories of the Arcade,” encouraging participants to share more personal life stories related to arcades and arcade games, such as arcade game playing experiences, social interactions in arcades, and the relationship between arcades and childhood.

At the beginning of each interview, information on the study was presented. Participation was voluntary and anonymous, and data were anonymized and stored securely in compliance with GDPR and institutional protocols. All interviews were approved by ethical review and written consent from participants, during the process, we adhered to the principles of self-reflexive, collaborative, and participatory to identify possible biases by reflecting on who we are and our research motivations (Murdock and Pink, 2005). To minimize ethical risks, the interview prompts were worded carefully to avoid distressing language and participants were encouraged to guide the depth and direction of their contributions.

The semi-structured interviews revolved around themes such as players’ experience of playing arcade, their favorite games, feelings toward arcade, social interactions in arcade halls, and childhood memories associated with arcade. It comprised three sequential rounds: First, we inquired about participants’ first experience of playing arcade, including motivations for playing arcade games, duration, game selections, and rankings. Then, we encouraged respondents to recall the stories that took place in the arcade hall. Finally, we invited respondents to express their views on arcade and summarize the group cultural identity they have established through playing arcade games. Followed by a sequence of questions: (1) Can you recall your first experience in the arcade hall? (2) What arcade game do you like best? (3) How do you feel about playing this game? (4) Do you miss the game time in the arcade hall? (5) Do you still go to the mall to play arcade games now? What’s your opinion on the current arcade games? The questions were adapted to each person’s level of verbal comprehension and expression. This study adopts a qualitative interview approach, aiming to deeply explore the meaning-making mechanisms and experiential world of the nostalgic practices among China’s first-generation arcade players, rather than pursuing statistical representativeness. We acknowledge that the 18 interviewees recruited through specific communities provide an in-depth understanding of this phenomenon, rather than universally applicable conclusions. Although interview results cannot be generalized to represent every player’s experiences within the player community, analyzing multiple interviews can reveal shared experiences, stories, or thoughts among players, thus offering a comprehensive understanding of collective memory in gaming.

With the assistance of MaxQda software, our analysis followed the pathway outlined below: First, through open coding, we extracted 133 initial concepts from the interview transcripts, such as “the dazzling appearance of the arcade cabinet,” “the cheers of onlookers,” “heading straight to the arcade after school,” and “the pain in my fingers.” Subsequently, during the axial coding phase, we grouped these concepts into more abstract subcategories, such as “mechanic experience,” “the materiality of the machine,” “embodied interaction” and “spatial atmosphere.” Finally, in the selective coding phase, centered on the core category of “nostalgic practice,” we integrated these subcategories into three interrelated dimensions: virtual world nostalgia, machine nostalgia, and spatial nostalgia. This process constructed the interactive memory framework for this paper (Table 1). The first generation of gamers in China, through the narrative of arcade game memories, demonstrated the energy and emotions they invested in the arcade halls, how arcade games influenced their daily lives, and the important role they played in their life courses. The memories constructed in the entanglement of arcade halls and childhood life contain profound emotional value and social significance, constituting an important dimension for exploring the narrative production of individuals, as well as society, the nation, and culture.

**TABLE 1:** Three-level coding process of game nostalgia.

Selective coding (core categories)	Axial coding (main categories/subcategories)	Open coding (representative interview excerpts)
A Virtual world nostalgia (text interaction)	A1 Game text memory • A1a Characters and plots • A1b Visuals and levels	The main character Cody in white, the red-suited Guy, and the fat police officer–these three were rescuing the daughter of the police chief from a shadowy gang. (L1)
	A2 Mechanic experience memory • A2a Difficulty and challenge • A2b Sense of achievement and frustration	If you were really good, one coin could last you the whole afternoon. (D1) It satisfies my curiosity and desire to finally defeat the boss I couldn’t reach back then. (Z2)
	A3 Cross-platform compensation • A3a Emulator experience • A3b Compensation for unfinished games	Now the mindset is different. There are too many games nowadays, and that sense of curiosity is gone. (X1)
B Machine nostalgia (human-computer interaction)	B1 Materiality of the machine • B1a Appearance and lighting • B1b Joysticks and buttons	The arcade was very dim, but the machines were so dazzling–full of colors. There was a special “Black PS2” room with four huge “big-butt” TVs, each connected to a PS2. (G1)
	B2 Embodied interaction • B2a Pain in fingers • B2b Bodily memory of operation	My youth was spent furiously pressing buttons in the arcade. Even now, when I think of it, my fingers still feel the pain. (G2)
	B3 Collection and disappearance of objects • B3a Collection of game tokens • B3b Disappearance and museumification of machines	Later, many arcades closed down, and the machines vanished, but I’ve kept a large collection of arcade coins. Back in the 1990s, I was a real “tycoon” in the arcade hall. (L2)
C Spatial nostalgia (interpersonal interaction)	C1 Spatial atmosphere • C1a Dimness and noise • C1b Hidden locations	Most arcades back then were set up near wet markets, street-side shops, or dark alleys. The locations were quite hidden: you usually needed someone to take you there. (Y2)
	C2 Public life and social interaction • C2a Watching and cheering • C2b Conflict and chaos	The place was smoky and full of swearing, but when someone played well, people would cheer; it was a fascinating experience. (W1) Early arcades were full of smoke. There was also the “Young and Dangerous” culture–idle youths hanging out, often getting into fights or stealing coins. (K1)
	C3 Emotional infrastructure • C3a Weak-tie sociality • C3b Emotional solace and belonging	The kids who went to play games often ended up getting scolded together when they got back. (K1) Many of the friends I could talk with were made there while playing games. Back then, only games could heal me. (G1)

## 4 An interactive memory framework for game nostalgia

In the following section, this paper presents empirical cases of players’ arcade nostalgia. Following Menke’s practice-oriented view of media nostalgia, particularly the notion that nostalgic practice is realized only through the body and retrospective reflection of the user, and in conjunction with interview materials, we find that players have constructed a model of game nostalgia practice grounded in bodily interaction. During their engagement with arcade games, players employ three primary modes of subjective action: manipulating avatars within the virtual world, engaging in human-computer interaction with the arcade game machine, and participating in interpersonal interactions within the arcade hall. These three forms of interaction are mediated by the player’s bodily engagement with the arcade machine. This process generates nostalgia of the arcade machine itself, which then extend to nostalgia of the game text within the virtual world and nostalgia of the arcade hall space. This culminates in the formation of an interactive memory framework comprising virtual world nostalgia, machine nostalgia, and spatial nostalgia.

### 4.1 Text interaction: nostalgia for virtual worlds

First, arcade nostalgia manifests through the recollection of specific periods of gaming texts, including nostalgic memories of game genres, characters, and symbols. As one participant, L1 (October 13, 2024), recalled:

“The most impressive game was *Final Fight*. The main character Cody in white, the red-suited Guy, and the fat police officer–these three were rescuing the daughter of the police chief from a shadowy gang.”

The characters, level designs, and visual effects of arcade games serve as memory markers that trigger emotional attachment. Through revisiting these virtual worlds, players construct tangible and visualized memories of the game text itself. Furthermore, interactive features such as score displays, ranking systems, and coin-operated mechanisms function that structure memory practices. As participant D1 (September 22, 2024) recalled:

“Around 80% of players in the arcade were playing *The King of Fighters*. By around 1997, almost everyone in the arcade was dueling. If you were really good, one coin could last you the whole afternoon.”

Arcade gaming produced a highly immersive media environment that rewarded attention, focus, and emotion. The memory of arcade games thus carries layers of joy, excitement, challenge, competition, and frustration–emotions woven into the collective coming-of-age experiences of the player community. Such affective recollections allow players to revisit and re-experience previous states of life. When games migrate onto new platforms, the new textual quality often evoke memories of the past. On social media platforms such as Douban and Baidu Tieba, players collaborate in recollecting and reconstructing shared memories through posts like “Please help me find an old arcade game.” Participant Z2 (September 30, 2024) described this as an emotionally compensatory process: “Especially those games I never finished when I was young–it satisfies my curiosity and desire to finally defeat the boss I couldn’t reach back then.”

With the rise of retro game consumption, classic arcade titles have been repackaged for consoles and mobile devices, representing both a response to and adaptation of evolving media technologies. As Sobchack (2000) notes, transformations in technological forms and modes of interaction can elicit nostalgia affection. For instance, X1 (October 10, 2024) mentioned playing old arcade games on a “Arcade Emulator” but reflected: “Now the mindset is different. There are too many games nowadays, and that sense of curiosity is gone.” Rather than erasing arcade gaming from memory, technological development has reinforced players’ identity grounded in the arcade text. Watching, collecting, and emulating old arcade games enables players to reconnect with past temporalities and spaces. As Wertsch (2002, p. 53) suggests, a community’s sense of shared past depends on its common textual resources–such as similar game genres, visuals, characters, and narratives. Nostalgia for arcade game texts, therefore, is not merely a cue of another time and place, but an expression of longing for a prior mode of existence and a digital homeland. By relaunching arcade games through new media technologies and platforms, players symbolically return to the same virtual spaces they once inhabited, experiencing a form of nostalgia that cannot easily be replicated in non-virtual contexts.

### 4.2 Human-computer interaction: technological nostalgia of arcade machines

The iteration of gaming machine has fundamentally transformed the way humans play. The action of gaming is built upon interactions between humans and machines, and through these interactions, people also establish relationships with one another. Technological nostalgia refers to an affective longing for or idealization of obsolete technologies–a memory practice that re-creates and re-imagines the images, sounds, and aesthetics of a bygone era (Campopiano, 2014). Such practices form an autobiographical narrative and public presentation of a generation’s social life history. As a material medium, the arcade machine plays an indispensable reconstructive role in the memory practices of the arcade era.

First, the arcade machine functions as the material infrastructure and medium of the simulation system: long-term human–machine interaction deepen the object’s emotional value and the sense of connection and presence between humans and things (Zhao, 2024). The material features of arcade cabinets–bright door frames, dynamic enclosures, rails, joysticks, and buttons–frequently appear in players’ narratives of memory. As Player G1 (October 26, 2024) recalled his first visit to an arcade: “The arcade was very dim, but the machines were so dazzling–full of colors. There was a special “Black PS2” room with four huge “big-butt” TVs, each connected to a PS2.”

With the rapid iteration of media technologies, arcade machines from the 1990s quickly disappeared from the market and became objects of nostalgia. At the moment when new technologies announce the end of old ones, a certain poetic quality of machines emerges (Schivelbusch, 2014). The life stories and cultural biographies that technological apparatus accumulate during their lifecycles reveal the emotional values attached to obsolete technologies amid social transformation. As Player L2 (November 2, 2024) noted, he collects arcade tokens to memorialize his past life in the arcade era: “Later, many arcades closed down, and the machines vanished, but I’ve kept a large collection of arcade coins. Back in the 1990s, I was a real “tycoon” in the arcade hall.”

The accelerated pace of social change generates instability and insecurity, which in turn fuels emotional yearning for old technologies (Menke, 2017). Through processes of collecting, displaying, preserving, and recalling, the social life of media artifacts–such as arcade coins–is extended (Zhao and Fung, 2025).

In recent years, the *China Audio-Video and Digital Publishing Association Game Museum (CADPA)* and various “retro arcades” have collected and exhibited gaming machines produced by *Sega*, *Capcom*, *SNK*, and *Tongli*. These old machines serve as windows into the history of gaming culture and as symbols of a bygone time. The act of collecting and displaying these machines represents a way to reclaim materiality and reflect on social and cultural transformations. For the “first-generation” players, arcade machines, as human-made media apparatuses, demonstrating a processual feature of transcending the sliding relationship between objects and symbols, and is a signifier with a connotation and an emotional evocation structure. The disappearance of arcade machines signifies not only the retreat of a material object but also the closure of a life phase. Players’ technological nostalgia thus constitutes a semiotic act that summons the return of the signifier, through which arcade machines regain meaning and value via recalling the past time.

Furthermore, the construction of memory is inseparable from patterns of interpersonal interaction. The meaning of a machine does not reside in the machine *per se*, but in what people do with it (McLuhan, 1994). Prezioso and Alessandroni (2022) conceptualize memory as a way of participating through things, an active, extended, semiotic, and situational engagement. In nostalgic practices of arcade players, recovering the feeling of the games they once played becomes crucial, an interviewee said: “My youth was spent furiously pressing buttons in the arcade. Even now, when I think of it, my fingers still feel the pain.” (G2, September 21, 2024).

Through the tactile memory of joystick manipulation, players encounter the machine through haptic intimacy. As the center of action in the game world, their interpretation of meaning arises from embodied, interactive experiences (Anable, 2018, p.39). Moreover, cognitive practices are also determined by the forms that things take (Gosden, 2005). Arcade cabinets structured modes of interaction centered on competitive gameplay, action-oriented themes, and the tactile knowledge of controllers. The longer players spent in the arcade, the more influence they gained within its competitive social environment. Nostalgia for arcades, therefore, also manifests as a form of competitive effort: through frantic button-mashing and joystick-spinning, players sought to reclaim authority, mastery, and cultural capital.

The arcade machine thus served as a material engine that shaped the everyday scenes and leisure culture of the 1990s. Its habituating mechanisms and routinized forms of human–machine interaction fostered a shared bodily experience among players. These ludic actions, grounded in interaction, constituted symbolic practices of meaning-making that not only invited collective memory of the machine’s physicality but also animated the emotional and cultural continuities between humans and media technologies.

### 4.3 Interpersonal interaction: spatial nostalgia of the arcade hall

The spatial location of the arcade, together with the colors, textures, and functions of the machines and the presence of players, collectively shape the affective structure of atmosphere. Atmosphere refers to a certain emotional quality of space, one that restores perception to the embodied sensations it provokes within an affective environment (Griffero and Tedeschini, 2019, p.2). The atmospheric composition of the arcade–its dim and hidden locations, the frenetic rhythm and noise of the games, the mingling of adult and child players, and the cheering onlookers–awakens among players with shared spatial memories a nostalgic recollection of their vanished childhood scenes. As interviewee Y2 recalled:

“Most arcades back then were set up near wet markets, street-side shops, or dark alleys. The locations were quite hidden: you usually needed someone to take you there. Near the crossroads by my house there was a huge arcade, and inside was a four-player machine for The King of Dragons. It felt incredible. The place was always packed, people queued up to play, and sometimes even fought for spots.” (Y2, September 30, 2024)

The reproduction of spatial meaning involves the interweaving of political, economic, cultural, and affective dimensions, shaping the public’s emotional experience and historical imagination of space. Within the arcade, players experienced the full ambience of arcade culture. Each machine acted as a mediating device that connected the real and virtual worlds while simultaneously dividing their boundary–drawing non-players and onlookers into the gaming situation and thus transforming them into participants within a shared public space. The essence of the arcade experience, therefore, lies in the player’s submission to the unpredictable and public nature of play (Harper, 2013).

Simultaneously, arcades were also sites of temptation and chaos, haunted by memories of gambling, bullying, and excessive noise. As player K1 (December 7, 2024) recalled: “Early arcades were full of smoke. There was also the “Young and Dangerous” culture–idle youths hanging out, often getting into fights or stealing coins.” This ambivalent atmosphere was closely tied to the arcade’s commercial nature as a profit-making machine. Z1 (November 16, 2024), a former arcade owner in Ningxia, recounted:

“I opened an arcade mainly to make quick money. Back then, most of the profits came from gambling machines–fishing games, slot machines–while other machines were just there to attract people. But it didn’t last long. The authorities cracked down hard, and my license was even confiscated.”

Different venues cultivate corresponding quality of place that imbue their environments with emotions and shape individual behaviors. Nostalgia for arcades thus also reflects broader social anxieties of the time–public concern over youth exposure to commercial entertainment–as well as the economic and cultural transformations and tensions brought by media technology.

Different venues cultivate corresponding quality of place that imbue their environments with emotions and shape individual behaviors. In May 2000, the Chinese news media Guangming Daily published an in-depth report titled “Video Games: “Spiritual opium” Targeting Children.” The article provided a detailed account of an undercover investigation into arcade halls, claiming that children who spent all their time in game rooms would end up one way only: boys would eventually become robbers and thieves, while girls would become hostesses in karaoke bars or prostitution. From then on, video games were thoroughly stigmatized with the label “Spiritual opium” in China. Nostalgia for arcades thus also reflects broader social anxieties of the time–public concern over youth exposure to commercial entertainment–as well as the economic and cultural transformations and tensions brought by media technology.

The arcade was also a social and affective space where interpersonal relations intertwined with entertainment and growth. All interviewees described arcades as public spaces of sociability. Nostalgia for arcades is always embedded in collective interactions–playing cooperatively, competing, sharing strategies and experiences, or even quarreling and fighting; onlookers cheering and commenting; and operators maintaining order. As players described: “In the arcade, machines were all grouped together. The place was smoky and full of swearing, but when someone played well, people would cheer: it was a fascinating experience.” (W1, November 3, 2024)

Through observing others in the public gaming environment, players not only learned how to play but also absorbed others’ ways of thinking and strategizing. The arcade gathered diverse social actors who collectively participated in the rewriting of arcade meaning. As a public space, the arcade included not only players but also onlookers, operators, and regulators–actors who directly or indirectly participated in the interaction and thus shaped the relationship between gameplay and sociability. The presence of onlookers and their relative skill levels enhanced the entertainment and performative qualities of play. These multiple, fluid roles were essential in constructing an interactive framework of memory, forming a distinctive memory landscape of the arcade hall.

## 5 The never-fading childhood and the arcade community of memory

Memory is both personal and collective–it functions as an individual response to lived experience while simultaneously constituting a shared reflection on social change. As Halbwachs (1992) reminds us, individual and collective memory form a closed circuit: memories are never purely private, but are sustained and reactivated through social frameworks. Collective memory involves not only the shared experiences of a group but also the narratives that corresponds to the past. Within media culture, shared recollections of outdated technologies or bygone eras become collective events that connect individuals to friends, families, and broader cultural imaginaries. It should be noted that the interviewees in this study were primarily recruited from active online nostalgia communities. Their memory narratives may carry a stronger tone of “curated nostalgia,” which offers a unique perspective for understanding the constructed nature of memory, but also implies that their experiences may differ from those of the broader player community not active in such online groups.

### 5.1 From individual to collective: the interwoven life of the neighborhood

The arcade game, as a specific media form, is a crucial site where personal and collective memories intertwine. Embedded in the technological transitions of the 1980s and 1990s, arcade gaming both witnessed and symbolized the fleeting nature of childhood under modernization. The memories associated with arcades constitute a community of memory structured around the intertwined themes of neighborhood life, generational identity, and nostalgia. This community is not merely a recollection of the past, but an ongoing articulation of cultural identity and social belonging. Through such articulation, the arcade’s media memory transcends the boundaries between the individual and the collective, technology and emotion, and past and present.

Data from player interviews demonstrate that arcade memory is deeply embedded in specific local spaces and social networks. Participants frequently referenced factories, neighborhoods, and residential compounds–spatial markers of 1990s urban life in China. Player Y2 (September 30, 2024) stated, “Back then, staff turnover wasn’t as high, and arcades had a strong local character. Basically, the people playing there were all from the neighborhood. After a while, as you saw each other more often, you naturally got to know one another.” Player K1 (December 7, 2024) recalled, “We were all kids from the same factory compound. It wasn’t like today, where boundaries are stronger. Back then, as compound kids, we had good neighborly relations. The kids who went to play games often ended up getting scolded together when they got back.” The arcade hall thus operated not simply as a site of leisure but as a public arena of everyday social interaction. As players recalled, the memories of gaming were inseparable from memories of friendship, rivalry, and cooperation. These micro-interactions collectively composed the texture of community life. In Halbwachs’s (1992) terms, memory persists only through such social frameworks: it gains meaning when individual recollection is validated by collective recognition.

Consequently, the arcade became a geo-social infrastructure through which players developed a sense of belonging and emotional support. Under the conditions of rapid urbanization and shifting family structures, arcades functioned as emotional refuges for many children and adolescents. An interviewee said: “When I was a child, I left my rural hometown to study in a county town in Gansu. I had no friends there and had poor relationships with my relatives. I went to the county town’s arcade hall at least once a week. Many of the friends I could talk with were made there while playing games. Back then, only games could heal me.”(G1, October 26, 2024) G1’s recollection profoundly reveals the role of the arcade hall as a form of “emotional infrastructure.” Against the backdrop of migration from rural communities to urban areas, it provided a temporary social anchor constituted by weak ties. This integration of place-based and interest-based connections serves as a micro-level manifestation of what Halbwachs describes as the social framework of collective memory.

From a media-sociological perspective, the collective memory of the arcade reflects the broader transformation from an embedded society to a disembedded one (Giddens, 1990). The nostalgia expressed by contemporary players is less about the machines per se and more about the thick sociality once afforded by shared physical spaces and face-to-face interactions. Therefore, nostalgia for the arcade signifies not only the reproduction of media memory but also a form of social critique–a response to the fragmentation and mobility anxieties of the present.

### 5.2 Generational memory and emotional structure

The study also highlights the generational dimension of media memory. For Chinese players born in the 1980s and 1990s, the arcade has become a generational symbol that transcends entertainment to embody a shared temporal and emotional landscape. Participants often referred to their experiences using expressions like “our generation” or “people of that time,” consciously situating themselves within a collective identity anchored in the past. Players Q1 (October 19, 2024) and Y1 (November 3, 2024) revisited the collective life of the 1990s through their memories of playing arcade games. “For those born in the 80s and 90s, the only form of entertainment back then was rushing to the arcade hall with our schoolbags on our backs after class. That’s a memory unique to our generation.” “People from our generation basically all have memories of sneaking off to the arcade after school and being dragged home by our parents for a spanking. Nowadays, attitudes toward gaming are changing, and arcades in shopping malls have become a place for parent-child activities.” Q1 and Y1’s statement, “That’s a memory unique to our generation,” directly corroborates Bolin’s (2017) thesis on “media generations.” However, it must be noted that this sense of generational identity, among the interviewees in this study, is often intertwined with a longing for “innocence” and an “acquaintance society.” Rather than an objective generational demarcation, it constitutes an emotional community constructed through nostalgic narratives. Through the materiality of its cabinets, the sensory tactility of its joysticks, and the co-present dynamics of its play spaces, the arcade mediated both time consciousness and social awareness among players. It thus produced a shared generational sensibility–a pre-digital form of media nativity preceding Prensky’s (2001) digital natives.

However, generational memory is not static. Rather, it constitutes a dynamic identity narrative, linking personal life stories with national modernization. The arcade’s associations with freedom, rebellion, and experimentation reflect how youth culture in reform-era China negotiated between collective discipline and emergent individualism. China’s first-generation players, who grew up with arcade games, have now become the backbone of contemporary society. This shift in social roles has facilitated a transfer of discursive power, which is key to destigmatization. As these first-generation players become parents, teachers, media professionals, or policymakers, their understanding of games tends to be more diverse and inclusive. Their own life experiences have demonstrated that “playing games does not equal moral degeneration,” thereby dismantling, at the generational level, the extreme accusation of games being “spiritual opium.” Furthermore, nostalgic cultural capital influences the current production of gaming culture. When collective memories of and nostalgia for arcades serve as an emotional bond and are transformed into films or cultural and creative products, games are elevated from “spiritual opium” to a cultural symbol of a generation. In this sense, the memory of arcade play is not only an emotional artifact but also a cultural expression of social transformation.

Players’ narratives repeatedly invoke sentiments of lost innocence and social alienation. When participants recall the old time, they often mention the changes that iteration of technology makes to people’s relationships. As player W1 (September 21, 2024) put it, “People back then felt more innocent, I think. We mostly played games offline, and arcades brought a completely different kind of feeling.” Player L3 (October 26, 2024) also emphasized the generational context of gaming: “I guess that’s just what made our era special. Times have changed, our senses have changed, people have changed. It used to be a society of acquaintances; now it’s a society of strangers.” Such reflections index a collective emotional structure where nostalgia becomes a response to perceived discontinuities between past and present. Nostalgia, therefore, functions as a cultural practice of temporal mediation, enabling players to make sense of social transformation through affective continuity. The arcade, as an emotional infrastructure, allows individuals to sustain coherence in identity amid the flux of digital modernity.

### 5.3 Nostalgic consumption among contemporary arcade players

“In the pedestrian scenic area near my home, there is a row of such arcade machines. On each machine is a sticker that reads: “The arcade games we played back in the day–they were the little me, they held my entire childhood.” From children to middle-aged people, everyone loves to come here and play. But most of the machines have already broken down from overuse; now there’s only this one left.” (P1, October 13, 2024)

In the contemporary media environment, arcade memory is reactivated through nostalgia consumption and the cyclical reproduction of media heritage. The revival of retro aesthetics in urban consumer culture–arcade-themed venues in malls, commemorative posters, and reissued games on Steam or PlayStation–illustrates how the collective memory of arcades is commodified and circulated. On short-video platforms like *Kuaishou*, clips of middle-aged players revisiting *Three Kingdoms* games have generated millions of views, evidencing how arcade nostalgia bridges generations through mediated affect. Nostalgia consumption represents what Bolin (2014) calls a bittersweet passion: a symbolic and affective practice that reestablishes emotional continuity with the past self. The act of replaying old games, purchasing tokens, or visiting retro arcades allows players to materialize memory through ritualized engagement. Such acts of consumption simultaneously produce emotional satisfaction and reaffirm cultural identity.

However, the commercialization of nostalgia also entails a paradox. As arcades are recontextualized into sanitized, family-friendly spaces within shopping complexes, the gritty, subcultural vitality of the original street arcades is often erased. Memory becomes decontextualized, transformed from a site of lived social experience into a depoliticized aesthetic commodity. In this process, nostalgia is neutralized as sentiment rather than mobilized as critique. The re-emergence of arcade culture thus illustrates not a simple continuation but a reconfiguration of collective memory under the logic of capital and media reproduction. “The arcade games we played back in the day–they were the little me, they held my entire childhood.” This slogan printed on the machines perfectly captures the essence of contemporary nostalgic consumption: it offers carefully curated, consumable emotional symbols. The interviewees in this study, as active members of nostalgia communities, their memory narratives themselves participate in the production and circulation of this curated nostalgia. Their recollections are not merely a restoration of the past, but rather a performance and re-creation of it, serving to fulfill the present need for emotional continuity and identity. The persistence of arcade memory reveals the intricate entanglement of media, society, and emotion. As a form of media heritage, the arcade encapsulates both the technological modernity and the social transformation of post-reform China. Through the remembrance of arcade play, players reassemble fragments of childhood, reestablish social bonds, and reconnect with the temporalities of their youth.

Ultimately, the arcade functions as a memory device–a mediating structure through which collective experience circulates across generations. It enables the re-enchantment of the everyday by embedding affect, narrative, and ritual into the practice of play. The endurance of arcade nostalgia lies not in its replication of the past but in its capacity to symbolically repair the ruptures of the present. In this way, the “never-fading childhood” of arcade memory stands as a cultural testament to how play, technology, and remembrance merge in the ongoing negotiation between modernity and belonging.

## 6 Discussion and conclusion

The accelerated evolution of digital technology and media forms has intensified the postmodern subject’s emotional longing for past eras. Nostalgic memory, as a symbolic means of resisting the eternal present, facilitates an understanding of how media experiences from earlier periods shape contemporary subjects’ emotional responses, social imaginaries, and actions within today’s technological and cultural contexts. Video games, born alongside technological transformation, possess profound memory significance. They not only offer insight into the development of the gaming industry but also stand as witnesses to the growth of a generation, embodying affective, social, cognitive, and cultural dimensions. Research on the media memory of games has become a continually expanding field, addressing questions of how media technologies shape collective culture, construct meanings, and influence people’s understanding of themselves.

Arcade games are socially embedded, as both sensory environment and cultural site that gives rise to broader political, discursive, and expressive practices. The historical trajectory of arcades as a cultural form demonstrates the evolution of faith in media technologies throughout the digital age. Examining how the “first-generation” players narrate their memories of arcades–how they remember, and what forms of public life, generational experience, and collective emotions are invoked through such remembrance–helps to elucidate their understanding of their generational media identity. It further reveals how their past media experiences inform the affective and cultural dimensions of contemporary game consumption. Focusing on players who came of age during China’s *reform and opening-up* period also provides a lens through which to observe the rapid interplay between globalization and localization in the 1990s, as well as the concurrent transformations in media technology, commodity culture, popular aesthetics, and social life.

The memory narratives of first-generation players form a 3D framework encompassing game text, material technology, and spatial structure. Memories of game texts are manifested through players’ narrative reconstructions of scenes, avatars, and plots–revisiting virtual worlds as a way to re-materialize and visualize their past experiences. As a media machine, the arcade cabinet functions as a meaningful signifier and an affective trigger: through recalling embodied interactions with the joystick and buttons, players recover the tactile and material sensations of arcade play. The arcade hall, characterized by its hidden publicness and sociality, brought together players, onlookers, and operators, enriching the dimensions of mnemonic writing and forming the aura of arcade space.

Moreover, the media memory sustained by arcade play manifests as a community of memory centered on weakly connected neighborhood life and generational identity. Such communal memory provides a narrative resource and interpretive framework for players to construct meaning and identity, fostering collective self-description while consolidating and extending their cultural belonging. In today’s highly mobile and story-saturated consumer environment, arcade memory is reactivated through the mode of nostalgia consumption. By “paying for sentiment,” players cultivate a longing for the past and endow arcade media with renewed affective value, granting them new life within the retro-cultural revival.

The rise and decline of arcades in the 1990s mirrored social anxieties surrounding youth exposure to commercial entertainment, as well as broader concerns about emerging technologies and cultural-economic transitions. Their recent revival in the realm of consumption reveals a dual phenomenon: on the one hand, a response to the accelerated tempo of technological development and the desire to slow down by reconnecting with the past; on the other hand, an attempt to relive and compensate for lost gaming time by consuming retro games as a means of exploring alternative identities. Players’ recollections of arcades constitute a process of meaning-making, interweaving personal life trajectories with collective identities, shared histories, and the rhythms of mass consumer culture.

As a media object, cultural practice, and affective structure, the arcade embodies collective desires, fears, and everyday temporalities within a networked, unstable, and procedurally generated world. Through the temporary sharing of nostalgic emotions, players find strength, alleviating negative affect through repeated acts of emotional consumption, and thereby sustaining their momentum in real life. The formative media experiences of youth are reproduced through the narratives of memory, maintaining a lifelong sense of identity continuity. In this sense, the arcade stands both as a modern monument and a postmodern map–pointing to the places players once inhabited and, symbolically, still dwell within today.

Despite the discoveries about game nostalgia in this study, it has some limitations. Since the research focuses on nostalgic memories, participants’ recollections are inevitably influenced by the passage of time, the discursive atmosphere of online communities, and personal emotions. This may lead to memory blurring, reconstruction, or even glorification; more importantly, the high engagement of community members in nostalgic discourse may result in “curated memories” rather than unfiltered, original recollections, which may reduce the objectivity of the data. Future research could focus on the variations of arcades in today’s urban spaces and the adaptations of arcade games, and researchers can expand the sample size and recruitment channels, including offline groups and non-online active populations, to improve the representativeness of the sample; meanwhile, combining qualitative interviews with quantitative methods and objective data can further verify the authenticity of the data and enhance the credibility and generalizability of the research conclusions.

## Data Availability

The original contributions presented in this study are included in this article/supplementary material, further inquiries can be directed to the corresponding author.
